# The Clonal Conundrum: Clonal CD4⁺ Cutaneous Lymphoid Infiltrate in a Toddler Along a Reactive Primary Cutaneous CD4⁺ Small/Medium T-cell Lymphoproliferative Disorder (PCSM-TCLPD) Spectrum

**DOI:** 10.7759/cureus.106199

**Published:** 2026-03-31

**Authors:** Sri Naidnur, Kelly Maedo, Sonia Amy Neave, Emily DeSantis, Rick Lin

**Affiliations:** 1 Dermatology, Oasis Dermatology Group, McAllen, USA; 2 Dermatology, HCA Healthcare Corpus Christi Medical Center-Bay Area Program, McAllen, USA; 3 Dermatopathology, Sagis Diagnostics, Houston, USA

**Keywords:** ctcl: cutaneous t-cell lymphoma, early childhood, pcsm-lpd, plaque, primary cutaneous cd4+ small/medium t-cell lymphoproliferative disorder (pcsm-lpd), toddler

## Abstract

Cutaneous lymphoid infiltrates in children pose a diagnostic challenge because reactive lymphoid processes and low-grade lymphoproliferative disorders can share overlapping clinical, histopathologic, and molecular features.

We report a case of a two-year-old child presenting with a persistent erythematous plaque on the right cheek following a presumed mosquito bite. The lesion was refractory to topical antibiotics and corticosteroids. Histopathologic examination revealed a dense CD4⁺ T-cell-predominant dermal infiltrate, and molecular studies demonstrated both beta and gamma T-cell receptor (TCR) clonality. Although the findings were histopathologically compatible with primary cutaneous CD4⁺ small/medium T-cell lymphoproliferative disorder (PCSM-TCLPD), the patient’s age, clinical context, and subsequent clinical course suggested an atypical clonal reactive T-cell process.

Following a diagnostic biopsy, the lesion demonstrated partial clinical regression. A conservative management strategy with clinical observation and serial photography was adopted, avoiding unnecessary systemic therapy. This case highlights the diagnostic gray zone between clonal reactive lymphoid proliferations and PCSM-TCLPD in very young children, an age group in which such presentations are exceedingly rare, and emphasizes the importance of clinicopathologic correlation, multidisciplinary evaluation, and cautious management. It also raises awareness that these entities may exist along a biological spectrum rather than representing entirely distinct processes.

## Introduction

Cutaneous lymphoid infiltrates span a broad spectrum, ranging from benign reactive processes to indolent lymphoproliferative disorders [1]. Within this spectrum, cutaneous lymphoid hyperplasia (CLH), also referred to as cutaneous pseudolymphoma, represents an exaggerated local immunologic response to antigenic stimuli such as arthropod bites, trauma, or medications [1]. First described by Spiegler in 1894, CLH may clinically and histopathologically mimic cutaneous lymphoma despite its benign course and typically presents as solitary or localized erythematous to violaceous papules or nodules, most commonly on the head, neck, or upper extremities [1].

Histopathologically, CLH demonstrates a heterogeneous lymphoid infiltrate that may resemble either B-cell or T-cell lymphoma [1]. Although usually polyclonal, clonal T-cell populations, particularly in pediatric patients, have been reported and may create diagnostic confusion with primary cutaneous CD4⁺ small/medium T-cell lymphoproliferative disorder (PCSM-TCLPD) [1-4].

PCSM-TCLPD is an uncommon entity characterized by a localized dermal proliferation of small- to medium-sized CD4⁺ T lymphocytes [2,4]. Historically classified as a cutaneous T-cell lymphoma, it was redefined in the 2018 World Health Organization-European Organization for Research and Treatment of Cancer (WHO-EORTC) classification as a lymphoproliferative disorder to reflect its indolent behavior and favorable prognosis [2]. It most often presents in middle-aged or older adults as a solitary lesion of the head or neck, with characteristic clinicopathologic, immunophenotypic, and molecular features that are interpreted in combination. These may include, among other features, expression of T-follicular helper (TFH)-associated markers (e.g., PD-1, BCL6, ICOS), which identify a subset of helper T cells involved in immune responses, and T-cell receptor (TCR) clonality (a molecular finding indicating expansion of a single T-cell population), both of which may be seen in reactive as well as lymphoproliferative conditions [2,4]. Although classically described in adults, rare cases have been reported in very young children [5,6]. Distinguishing CLH from PCSM-TCLPD can be particularly challenging in pediatric patients, where reactive processes are more common, and clonality does not necessarily indicate malignancy [1,3]. Reports in children younger than five remain limited, highlighting an important age-related diagnostic gap.

## Case presentation

**Figure 1:**
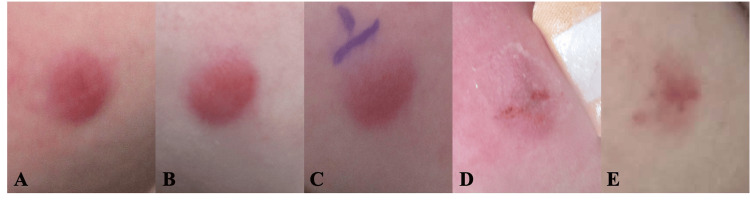
Clinical follow-up of the lesion over time (A) Approximately three weeks after lesion onset, showing a well-demarcated erythematous plaque on the right cheek (B) Seven weeks after lesion onset, with a persistent erythematous plaque (C) Ten weeks after lesion onset, with the site marked prior to punch biopsy (D) Twelve weeks after lesion onset (two weeks post-biopsy), demonstrating early lightening of the lesion following suture removal (E) Six months after lesion onset (approximately three months post-biopsy), demonstrating interval clinical regression with faint residual central erythema within a lighter erythematous patch

A previously healthy two-year-old male presented with a persistent erythematous lesion on the right cheek. The patient’s mother reported that the lesion appeared approximately three weeks after a presumed mosquito bite, gradually enlarged, and then stabilized in size (Figure 1A). The child was otherwise well, meeting developmental milestones and was up to date on vaccinations, though receiving speech therapy. There were no constitutional symptoms, including fever, weight loss, or night sweats.

Physical examination revealed a solitary, well-demarcated erythematous plaque measuring approximately 1.3 × 1.0 cm on the right cheek. The initial clinical differential diagnosis included mastocytoma, Spitz nevus, and juvenile xanthogranuloma. The lesion was initially treated with topical mupirocin, triamcinolone, and hydrocortisone for a presumed inflammatory or infectious process. However, no clinical improvement was observed (Figure 1B). Given the persistence of the lesion, a 4-mm punch biopsy was performed approximately six weeks after the initial presentation (Figure 1C). Figure 1A-1E illustrates the temporal clinical progression of the lesion from initial presentation through post-biopsy regression.

**Figure 2:**
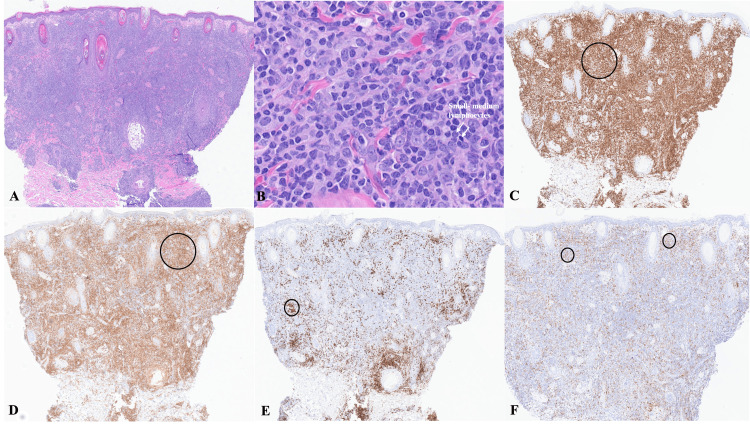
Histopathologic and immunohistochemical findings of the cutaneous lymphoid infiltrate (A) Low-power H&E demonstrating a dense dermal lymphoid infiltrate (×2 magnification) (B) Higher-power H&E showing predominance of small- to medium-sized lymphocytes (arrows) (×40 magnification) (C) CD3 highlighting a diffuse T-cell–predominant infiltrate (circle indicates positive staining) (D) CD4 demonstrating a predominance of CD4⁺ T lymphocytes (circle indicates positive staining) (E) CD20 highlighting scattered B lymphocytes (circle indicates positive cells) (F) ICOS positive in a subset of lymphocytes (circles indicate positive cells)

Histopathologic evaluation with hematoxylin and eosin (H&E) staining demonstrated a dense nodular lymphoid infiltrate composed of small-to-intermediate-sized lymphocytes extending from the superficial to deep dermis, with focal extension into the subcutis (Figures 2A, 2B). There was no significant epidermotropism, necrosis, or granulomatous inflammation. Immunohistochemical (IHC) studies (Figures 2C-2F) revealed a CD4-predominant T-cell infiltrate with scattered B cells and mixed inflammatory cells. The proliferative index was low (Ki-67 ~15%). The full IHC profile is summarized in Table 1. Molecular analysis by next-generation sequencing demonstrated monoclonal rearrangements of both beta and gamma TCRs, supporting an atypical clonal T-cell infiltrate. 

**Table 1:** Immunohistochemical profile of the lesion

Marker	Result	Interpretation
CD3, CD5, CD7, CD43	Positive	Pan–T-cell markers highlighting predominant T-cell infiltrate
CD4 : CD8 ratio	~1.5: 1	Mild CD4⁺ predominance
CD20	Scattered positive cells	Minor B-cell component
CD30	Scattered cells	Activated immunoblasts
S100, CD1a, Langerin	Scattered positive cells	Dendritic and Langerhans cells
CD68	Scattered positive cells	Histiocytes/macrophages
CD34	Positive in vascular structures	Background vasculature
Cytokeratin AE1/AE3	Negative	No epithelial involvement
CD56	Scattered positive cells	NK cells/cytotoxic lymphocytes
PD1, BCL6, ICOS	Positive in a subset of cells	T-follicular helper (TFH)–associated markers
CXCL13	Rare staining	Limited T-follicular helper expression
TCR βF1	Majority positive	Predominant αβ T-cell population
TCR δ	Minority positive	Small γδ T-cell population
Ki-67	~15%	Low proliferative index

IHC findings were assessed in keeping with routine dermatopathologic assessment. Select markers, including Ki-67 and the CD4:CD8 ratio, were reported with quantitative or proportional estimates, whereas TFH-associated markers (PD1, BCL6, ICOS) were not assigned precise percentages.

Dermatopathologic interpretation, in consultation with a hematopathologist, favored a low-grade PCSM-TCLPD. However, given the patient’s young age and preceding arthropod bite, an atypical clonal reactive lymphoid proliferation remained a significant consideration. Subsequent multidisciplinary review involving pediatric hematology-oncology and dermatopathology supported the interpretation of the findings as an atypical clonal CD4⁺ T-cell infiltrate, best viewed within a reactive-PCSM-TCLPD spectrum rather than a definitive PCSM-TCLPD.

The patient was referred to pediatric hematology-oncology for further evaluation. Laboratory testing showed a normal absolute lymphocyte count (3,479/µL) with otherwise unremarkable findings. Due to limited tissue for additional in situ studies, serologic testing for Epstein-Barr virus (EBV), cytomegalovirus (CMV), and human T-lymphotropic virus (HTLV-1/2) was performed and was negative. In the absence of systemic findings, imaging was deferred.

The diagnostic uncertainty initially caused parental concern, which improved following multidisciplinary evaluation and a reassuring clinical course. The lesion demonstrated gradual partial regression after biopsy, evolving from a well-demarcated plaque to a faint residual patch over six months (Figures 1D, 1E).

Given the benign course and multidisciplinary consensus, pediatric hematology-oncology recommended a conservative, watchful-waiting approach, with planned dermatologic and/or pediatric follow-up every three months during the first year and every six months during the second year, including clinical examination and photographic documentation at each visit.

Notably, the patient’s very young age and the presence of both clonal T-cell proliferation and features suggestive of a reactive process distinguish this case from classic adult presentations of PCSM-TCLPD and typical reactive CLH.

## Discussion

This case highlights key diagnostic challenges in evaluating cutaneous lymphoid infiltrates and underscores the importance of clinicopathologic correlation. Dermatopathologic evaluation demonstrated an atypical dermal CD4⁺ T-cell infiltrate with TFH marker expression and positive beta- and gamma-TCR gene rearrangements, favoring a low-grade PCSM-TCLPD. This interpretation was supported by small- to intermediate-sized lymphocytes with mild cytologic atypia, a modest CD4 predominance (CD4:CD8 ~1.5:1), and a polymorphous background infiltrate composed of scattered B cells, plasma cells, and histiocytes [1,3]. In PCSM-TCLPD, specific CD4:CD8 ratios are not uniformly defined [1,2]. However, the patient’s very young age and preceding arthropod bite raised concern for an atypical reactive process with clonality [1,3]. TCR clonality does not always indicate malignancy and may be seen in reactive CLH, although it is more frequently identified in PCSM-TCLPD [1-3]. The historical classification of PCSM-TCLPD as a lymphoma may also contribute to caregiver anxiety and unnecessary imaging or interventions despite its typically indolent course [2,4].

The biological significance of dual TCR beta and gamma clonality requires careful interpretation. TCR clonality reflects the expansion of T-cells with identical receptor gene rearrangements, indicating a shared cellular origin [1,3]. Detection of clonality in both TCR beta and gamma chains suggests expansion of a limited number of T-cell populations [1,3]. Although this may raise concern for a neoplastic process, clonality is not specific for malignancy [1,3]. In reactive conditions, particularly in children, antigenic stimuli, such as arthropod bites, infections, or trauma, can drive selective expansion of certain T-cell clones, resulting in detectable clonality [1,3]. Conversely, similar clonal patterns may also be seen in indolent lymphoproliferative disorders such as PCSM-TCLPD [2,4]. Therefore, clonality should be interpreted in the context of clinical features, histopathology, and disease course, rather than in isolation [1-4].

Multidisciplinary evaluation, including dermatopathology, hematopathology, and pediatric hematology/oncology, supported this diagnostic ambiguity. Although rare pediatric cases of PCSM-TCLPD have been reported [5,6], Table 2 highlights reported cases by age and anatomic location to illustrate that this entity can occur in very young children and across varied sites. Clonality and treatment details were not consistently reported. In one reported case involving a six-month-old female infant, a left cheek nodule was managed with surgical excision [5]. At that time (2015), this entity was classified as a lymphoma, which may explain the decision to perform excision. Under the 2018 WHO-EORTC classification, it is now considered a lymphoproliferative disorder [2]. In contrast, our case demonstrated spontaneous partial regression following biopsy and was managed conservatively with observation. Notably, regression following biopsy has been reported in both reactive cutaneous lymphoid proliferations and PCSM-TCLPD and therefore does not reliably distinguish between these entities. A focused literature search was performed using terms including “PCSM-TCLPD,” “pediatric,” “very young children,” and “≤5 years” to identify illustrative cases. Notably, some reported cases may also fall along a reactive-PCSM-TCLPD spectrum; however, limited data make it difficult to place them on this spectrum.

**Table 2:** Reported cases of PCSM-TCLPD in children ≤5 years – indicates data not reported or specified in the original publication PCSM-TCLPD: primary cutaneous CD4⁺ small/medium T-cell lymphoproliferative disorder

Age	Sex	Location	Clinical Morphology	Clonality	Management	Author/ Year
6 months	Female	Left cheek	Solitary nodule	Positive	Surgical excision	Li et al., 2015 [5]
6 months	Male	Left cheek	Solitary nodule	–	–	Odeuyan et al., 2025 [6]
3 years	Male	Right posterior thigh	Solitary macule	–	–	Odeuyan et al., 2025 [6]
5 years	Female	Right axillary region	Solitary plaque	–	–	Odeuyan et al., 2025 [6]
5 years	Female	Head	Solitary nodule	–	–	Odeuyan et al., 2025 [6]
5 years	Male	Chest	Solitary nodule	–	–	Odeuyan et al., 2025 [6]

Table 3 summarizes key clinicopathologic features of CLH and PCSM-TCLPD. The categorical differences are presented in a tabular format to facilitate quick understanding and visual reference, while providing clinical and histopathologic context. Although CLH is generally considered reactive and PCSM-TCLPD clonal, distinctions are not always clear. Both entities may also share similar antigenic triggers, including arthropod bites, infections, or trauma, further contributing to overlapping clinicopathologic features [7]. Overlapping features, including CD4⁺ predominance, similar clinical morphology, and occasional clonality, limit reliance on any single diagnostic criterion and reinforce the importance of integrated clinicopathologic correlation [1-3].

**Table 3:** Comparison of Reactive CLP and PCSM-TCLPD Adapted and synthesized from published literature [1-3]. PCSM-TCLPD: primary cutaneous CD4⁺ small/medium T-cell lymphoproliferative disorder

Feature	Reactive Cutaneous Lymphoid Proliferations	PCSM-TCLPD
Etiology	Antigen-driven immune response (arthropod bites, trauma, infection)	Clonal CD4⁺ T-cell lymphoproliferative disorder
Age group	More common in children	Most common in middle-aged adults
Clinical appearance	Often nonspecific papules, plaques, or nodules	Often nonspecific solitary papule, plaque, or nodule
Histopathology	Mixed inflammatory lymphoid infiltrate	Dense CD4⁺ T-cell–predominant infiltrate
TCR clonality	Usually polyclonal, though clonality may occasionally be detected	Frequently clonal
Clinical course	Often self-limited	Indolent localized disease
Regression	Spontaneous or after biopsy	Spontaneous or after biopsy reported
Prognosis	Excellent	Excellent

Overall, these findings are best interpreted as an atypical clonal CD4⁺ T-cell infiltrate along a reactive PCSM-TCLPD spectrum. While not a formal diagnostic category, this framework reflects real-world clinicopathologic overlap. Recognition of this overlap supports conservative management with close follow-up and helps avoid unnecessary interventions in otherwise indolent cases.

## Conclusions

This case supports a spectrum-based understanding of clonal CD4⁺ cutaneous lymphoid infiltrates rather than a strict binary classification. In this patient, the findings were not entirely consistent with a classic reactive process or PCSM-TCLPD, but instead fell within a diagnostic gray zone along a reactive PCSM-TCLPD spectrum. This spectrum refers to a continuum in which reactive cutaneous lymphoid proliferations may demonstrate clonal T-cell populations and partial immunophenotypic overlap with PCSM-TCLPD, without fulfilling all criteria for a definitive lymphoproliferative disorder. Importantly, this represents a practical interpretive framework rather than a definitive diagnosis. Recognizing this overlap can help avoid overdiagnosis and unnecessary intervention and supports a conservative, multidisciplinary approach with close follow-up to minimize patient morbidity and parental anxiety. This case also highlights the importance of integrating clinical context, patient age, and disease course when interpreting clonality in pediatric cutaneous lymphoid infiltrates. Greater awareness of this diagnostic gray zone may help guide more thoughtful evaluation and management of similar cases in very young children.
